# Jejunal torsion around the right ureter presenting as postoperative bowel obstruction: a case report

**DOI:** 10.1186/1752-1947-8-209

**Published:** 2014-06-19

**Authors:** HuseyinYuce Bircan, Bora Koc, Umit Ozcelik, Alp Demirag

**Affiliations:** 1 Department of Surgery, Baskent University, Faculty of Medicine, Istanbul Research Hospital, Oymacı sokak No:7 Altunizade, Istanbul, Turkey

**Keywords:** Radical hysterectomy, Cervical carcinoma, Bowel obstruction

## Abstract

**Introduction:**

Since abdominal radical hysterectomy was first described by Clark and Reis in 1895, it has been commonly used in the primary surgical treatment of carcinoma of the cervix. We report the case of a 45-year-old woman who was diagnosed with a small bowel obstruction due to jejunal torsion to her right ureter mimicking postoperative adhesion ileus.

**Case presentation:**

A 45-year-old Turkish woman was admitted to our emergency department with complaints of abdominal pain, constipation, nausea and vomiting. She had undergone an abdominal radical hysterectomy for cervical carcinoma three years earlier. Computed tomography scans revealed intestinal dilatation, a large amount of free fluid in the abdominal cavity and an area suspicious for jejunal perforation. Because of these radiological findings suggestive of obstruction and bowel ischemia, our patient underwent emergency surgery. Operative findings that showed a jejunal segment was turned around her right ureter so that it was mimicking a fibrous band.

**Conclusions:**

In this current case, we present the first determined complication of radical hysterectomy. According to our case report, surgical oncologists should be aware of this complication and review the surgical technique. It is considered that readaptation of the dorsolateral peritoneal layer after extended pelvic lymph node dissection resulted in fewer complications.

## Introduction

Carcinoma of the uterine cervix is the most common gynecologic malignant neoplasm worldwide [1,2]. The most common histological type of malignant cervical neoplasm is squamous cell carcinoma, which accounts for 80 to 90 percent of all cervical cancer. Adenocarcinomas only account for approximately 15 percent of malignant cervical tumors [1-4]. Although surgery and radiotherapy are equally effective for treatment of early cervical cancers, abdominal radical hysterectomy is one of the treatment options for early-stage cervical cancer. In many institutions, radiotherapy is less preferable due to unexpected complications and long-term consequences [5,6].

Since Clark and Reis described abdominal radical hysterectomy (ARH) for the first time in 1895, it has been commonly used in the primary surgical treatment of carcinoma of the cervix. ARH was then detailed, widely described and performed by Wertheim more than 100 years ago [7]. Radical hysterectomy involves the removal of the cervix, uterus and supporting tissues, with pelvic lymphadenectomy and removal of the upper third of the vagina. Also the procedure includes pelvic and paraaortic retroperitoneal lymph node dissection. The ureters should be identified and preserved during the procedure [8]. Common intraoperative complications of this procedure are bowel injury and bleeding. In addition to surgical complications in general, specific late complications of the procedure include micturition and sexual problems [9].

In this case report, we present the case of a patient admitted to our emergency department with a mechanic bowel obstruction because of jejunal torsion around her right ureter as a late complication of ARH.

## Case presentation

**Figure 1:**
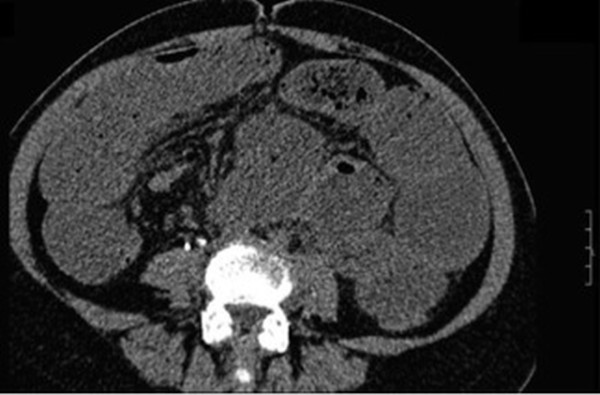
Computed tomography image: a group of poorly enhanced and thickened bowel walls on non-contrast computed tomography.

A 45-year-old Turkish woman was admitted to our emergency department with complaints of abdominal pain, constipation, nausea and vomiting. She had undergone abdominal radical hysterectomy for cervical carcinoma three years earlier. A physical examination revealed a serious abdominal distention, generalized abdominal tenderness and a rebound sign with a metallic bowel sound. Her blood pressure was 70/40mmHg, with a heart rate of 118 beats/min, a body temperature of 38.2°C and oxygen saturation of 92%. Laboratory tests showed a raised white cell count of 29.0×103/mmc, and a hemoglobin value of 14.0g/dL with a hematocrit level of 41.1%. The positive laboratory findings were C-reactive protein (CRP): 289mg/L, blood urea nitrogen (BUN): 56mg/dL, creatinine (Cr): 2.36mg/dL, sodium (Na):112mmol/L, potassium (K):2.7mmol/L. The arterial blood gas analysis revealed a lactic metabolic acidosis with a pH value of 6.99 and lactate level of 6.7mmol/L. In the historical examination, she had not been able to pass either gas or stool for three days. In her medical history, she had been admitted to the emergency room (ER) with a complaint of lower left abdominal quadrant pain but she had been discharged with a urinary tract infection diagnosis based on a urinalysis, which revealed pyuria with 56 leucocytes per mm^3^. An abdominal ultrasonography (US) scan was performed, followed by a computed tomography (CT) scan of her abdomen/pelvis. The abdominal US scan detected a swollen and immobile small bowel. The CT scan was performed for a better evaluation of our patient’s symptoms and confirmed the suspicion of mechanic bowel obstruction. The CT scan revealed intestinal dilatation, a large amount of free fluid in the abdominal cavity and an area suspicious for jejunal perforation. Moreover, the CT scan showed a group of poorly enhanced and thickened bowel walls (Figure 1).

**Figure 2:**
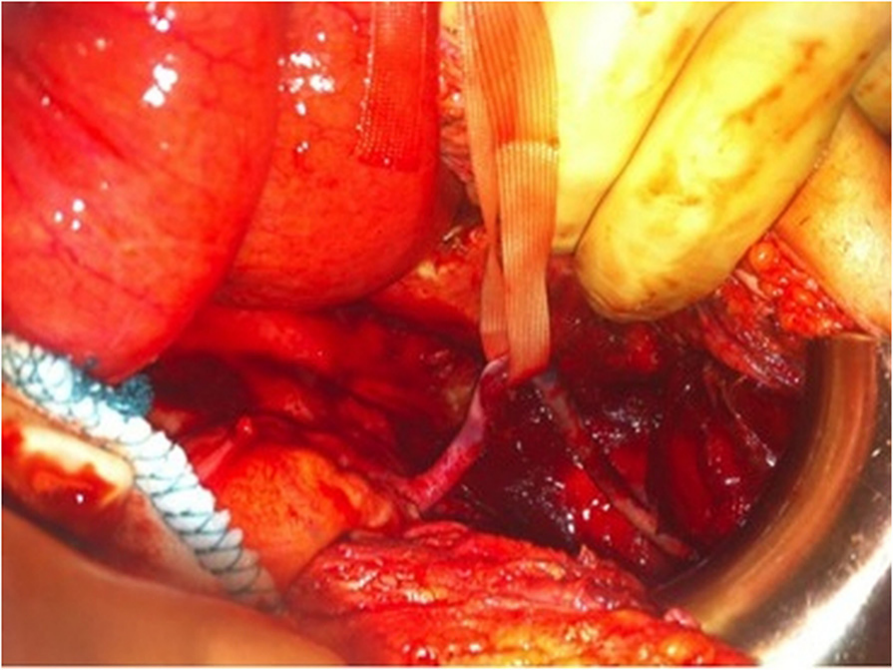
Operative image: the isolated right ureter and the scattered-ruined blood circulation of the ureter.

**Figure 3:**
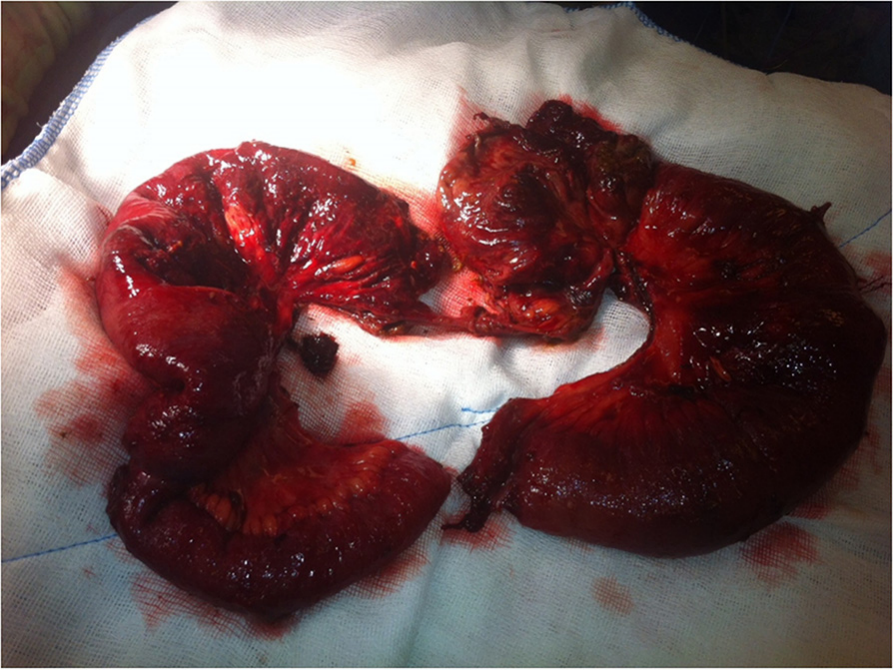
Specimen image: resected specimen of the small bowel due to perforation related to ureter torsion.

An aggressive fluid-electrolyte resuscitation (20ml/kg/hr) and antibiotics (ceftriaxone 2 gr/ 24h and metronidazole 500mg/6h intravenous) administration was started immediately in the intensive care unit. Because these radiological findings were suggestive for obstruction and bowel ischemia, our patient underwent emergency surgery six hours after admission.The prior midline incision was used to inspect the abdomen. The small intestines were dilated and there was a large amount of an exudative fluid in the abdomen. A jejunal segment was turned around a fibrous band and the surgeons realized that the ‘band’ was the right ureter (Figure 2). A 45cm jejenum resection and side-to-side jejunojejunostomy was performed. A suction drain was placed into the Douglas cavity (Figure 3).

She stayed in the intensive care unit until the postoperative second day. On the fourth day, she passed gas and started oral feeding with a high-calorie and high-protein diet. On the seventh day, she was given a normal diet. She developed a high body temperature (38.9°C). An abdominal CT scan was performed and a small amount of free fluid collection was detected between the small intestine loops. The fluid was aspirated at the interventional radiology unit. The fluid was clear and no microorganisms could be isolated in the fluid. She was discharged on the tenth postoperative day with normal oral feeding and excellent mobilization.

## Discussion

Radical hysterectomy and pelvic lymph node dissection have been considered as the standard surgical treatment for cervical cancer for more than 100 years [7]. In 1905, Wertheim reported a mortality rate of 18 percent and a major morbidity rate of 31 percent for the first 270 patients treated by radical hysterectomy [1,2,7-9]. Since that time, radical hysterectomy with pelvic lymphadenectomy has been performed with modifications in surgical technique as the major surgical treatment for cervical cancer. Improvements in surgical technique and advances in critical care medicine have resulted in lower operative morbidity associated with this procedure. Major urinary tract complications such as ureteral injury or vesicovaginal fistula are now extremely rare (<1%) [10].

Peritoneal adhesions may develop after pelvic and abdominal surgery or their combination [11]. Additionally, ingeminate pelvic or abdominal surgery increases the possibility of intra-abdominal adhesions. Retrospective studies have reported that 32 to 75 percent of patients who undergo abdominal reoperations encounter adhesion-related intestinal obstruction [12]. In contrast to congenital or postinflammatory adhesions, which are mostly asymptomatic, postoperative adhesions cause 40 percent of all cases of intestinal obstruction [13]. Intra-abdominal adhesions are predominantly diagnosed intraoperatively. Taking a careful history can substantiate the suspicion of adhesions; no other clinical investigations or imaging procedures enable a confident diagnosis [14]. In our case, we performed CT imaging due to the suspicion of a recurrent mass, but the CT images could not clarify the etiology of the bowel obstruction.

A urinary tract infection is a common diagnosis in ERs. However, pyuria is one of the important findings, for urinary tract infections may cause a misdiagnosis if the other causes are ignored. Retrocecal appendicitis or other closed organ infections may cause pyuria [15]. As reported in our case, a delayed or missed diagnosis may increase morbidity or mortality. A fully informed medical history and a thorough physical examination may be helpful for a proper diagnosis. It is well known that if there is no identified mass on radiologic images, an adhesion could be the cause of an obstruction. In our case, we considered a bowel obstruction due to adhesion and performed emergency surgery. The remarkable point of this case is the undefined cause of the mechanical bowel obstruction, which can be described as jejunal torsion around the ureter. As a result of extensive ureteral mobilization without reperitonealizing the ureters’ pelvic lymphadenectomy in radical hysterectomy, ureters uncovered with fatty tissue allow the bowel to twist around itself.

Many gynecologists advocate that closure of the posterior peritoneum is not necessary after an ARH, however, it may help to prevent retroperitoneal complications [16]. Janschek reported a study that suggested non-closure of the peritoneum on vaginal hysterectomy holds no risks and probably some advantages, for example faster resumption of bowel function. Additionally, no significant differences could be observed in analyzed surgical outcome and complications were similar whether the peritoneum was closed or not [17].

Roth *et al*. reported their results for frequent problems following extended pelvic lymph node dissection (PLND) and cystectomy with or without reperitonealization. Extensive adhesions between small bowel loops and the abdominal and pelvic walls or the iliac vessels are often found in patients who have to undergo a reoperation following PLND [18]. Small bowel adhesions may cause mechanical obstructions. Although adhesions between small bowel loops related to PLND have been described in the literature, there has not been any case of small bowel torsion around the ureter. Herein, the case that we have reported is the first case in the literature to report it after abdominal hysterectomy. The overclearing of the ureter may cause the small bowel to squeeze and create torsion around it. To the best of our knowledge, due to this unexpected complication of ARH, the posterior attachment of ureters may be preserved during the ARH procedure to prevent this kind of complication. Additionally, readaptation of the dorsolateral peritoneal layer after extended PLND resulted in fewer complications.

## Conclusions

Radical hysterectomy with lymphadenectomy still remains an important modality for gynecologic oncology procedures, keeping in mind both common and rare complications of the procedure may decrease delays in treatment and increase success in managing the complications. According to our case report, surgical oncologists should be aware of this complication and review the surgical technique. Closing the dorsolateral peritoneal layer after extended PLND is considered to result in fewer complications.

## Consent

Written informed consent was obtained from the patient for publication of this case report and any accompanying images. A copy of the written consent is available for review by the Editor-in-Chief of this journal.

## Abbreviations

ARH: abdominal radical hysterectomy; CT: computed tomography; ER: emergency room; PLND: pelvic lymph node dissection; US: ultrasonography.

## Competing interests

The authors declare that they have no competing interests.

## Authors’ contributions

HYB and BK took care of our patient and wrote the initial draft. HYB, BK, AD and UO operated on our patient. BK edited the manuscript and performed the literature review. All authors read and approved the final manuscript.
